# Movement Protein of *Cucumber Mosaic Virus* Associates with Apoplastic Ascorbate Oxidase

**DOI:** 10.1371/journal.pone.0163320

**Published:** 2016-09-26

**Authors:** Reenu Kumari, Surender Kumar, Lakhmir Singh, Vipin Hallan

**Affiliations:** 1 Plant Virology lab, CSIR-Institute of Himalayan Bioresource Technology, Palampur, 176061, Himachal Pradesh, India; 2 Academy of Scientific and Innovative Research (AcSIR), CSIR-Institute of Himalayan Bioresource Technology (CSIR-IHBT) Campus, Palampur, India; 3 Department of Biotechnology, Guru Nanak Dev University, Amritsar, 143005, India; 4 Department of Biotechnology, DAV University, Sarmastpur, Jalandhar, 144012, Punjab, India; National Institute of Plant Genome Research, INDIA

## Abstract

Plant viral movement proteins facilitate virion movement mainly through interaction with a number of factors from the host. We report the association of a cell wall localized ascorbate oxidase (CsAO4) from *Cucumis sativus* with the movement protein (MP) of *Cucumber mosaic virus* (CMV). This was identified first in a yeast two-hybrid screen and validated by *in vivo* pull down and bimolecular fluorescence complementation (BiFC) assays. The BiFC assay showed localization of the bimolecular complexes of these proteins around the cell wall periphery as punctate spots. The expression of *CsAO4* was induced during the initial infection period (up to 72 h) in CMV infected *Nicotiana benthamiana* plants. To functionally validate its role in viral spread, we analyzed the virus accumulation in *CsAO4* overexpressing *Arabidopsis thaliana* and transiently silenced *N*. *benthamiana* plants (through a *Tobacco rattle virus* vector). Overexpression had no evident effect on virus accumulation in upper non-inoculated leaves of transgenic lines in comparison to WT plants at 7 days post inoculation (dpi). However, knockdown resulted in reduced CMV accumulation in systemic (non-inoculated) leaves of *NbΔAO*-pTRV2 silenced plants as compared to TRV inoculated control plants at 5 dpi (up to 1.3 fold difference). In addition, functional validation supported the importance of AO in plant development. These findings suggest that AO and viral MP interaction helps in early viral movement; however, it had no major effect on viral accumulation after 7 dpi. This study suggests that initial induction of expression of AO on virus infection and its association with viral MP helps both towards targeting of the MP to the apoplast and disrupting formation of functional AO dimers for spread of virus to nearby cells, reducing the redox defense of the plant during initial stages of infection.

## Introduction

To establish successful infection in a susceptible host, viruses initially move from cell to cell followed by systemic movement through phloem sieve elements. Viral movement proteins (MPs) play a dynamic role in the spread of viruses by managing their transport through symplasmic connections i.e. plasmodesmata (PD). MPs mediate viral spread either in the form of virions or as viral ribonucleoprotein (vRNP) complexes (reviewed in [1–3]).

MPs modulate PD structure by increasing the size exclusion limit (SEL) through severing actin filaments or degradation of callose around PD, either directly or by employing host proteins to facilitate movement [4–8]. Viruses from the genus *Potexvirus* have their MPs organized as a triple gene block (TGB) and are speculated to target PD by endoplasmic reticulum (ER) association [9–11]. Various studies using the yeast two hybrid system and far western blotting revealed association of many host proteins with viral proteins: DNaJ family proteins of *Nicotiana tabacum* and *Arabidopsis thaliana* with the non-structural protein m (NSm) protein of *Tomato spotted wilt virus* (TSWV) [12]; multiprotein bridging factor 1 (MBF) from tobacco with MP of ToMV (*Tomato mosaic virus*) [13] and coat protein-interacting protein-L (IP-L) from tomato with the coat protein (CP) of ToMV [14]; pectin methylesterase (PME), calreticulin and ankyrin repeat containing protein from tobacco with MP of *Tobacco mosaic virus* (TMV) [8, 15, 16]; KELP from *A*. *thaliana* with MP of ToMV [17]; Rubisco small subunit from *N*. *benthamiana* [18] with the MP of ToMV; TGB protein1 of *Pepino mosaic virus* with Catalase 1 (CAT1) of tomato [19]; and TGB3 protein of *Alternanthera mosaic virus* with photo system II (PS II) oxygen evolving complex protein (PsbO) of *A*. *thaliana* and *N*. *benthamiana* [20]. The TMV MP not only endorsed movement of the virus but also allowed the spread of RNA silencing signal, an example of viral self-attenuation mechanism [21].

*Cucumber mosaic virus* has a single-stranded RNA (ssRNA) genome, belonging to the family *Bromoviridae* and the genus *Cucumovirus*. The virus is well known as one of the rapidly evolving pathogens with the largest host range (infecting 1200 plant species from 100 families). Considering its economic importance, CMV has been placed in the list of the “Top 10 Plant viruses in molecular plant pathology” [22]. The CMV MP facilitates the cell-to-cell movement of the virus by forming vRNP complexes supported with CP, where it shields the vRNP complex from degradation by RNases [23–25]. Deletion of 33 amino acids from the C-terminal region of the MP leads to CP-independent movement of the virus [26]. Previously, symptom variation by different CMV strains was also attributed to sequence changes in the RNA2 and MP regions [27]. CMV MP inhibited polymerization and severed actin filaments during *in vitro* studies and supported the view that MP induced the increase in SEL of PD [7].

Host specific responses of viruses prompted us to explore new plant targets to understand their mechanism of infection, which can subsequently be utilized to develop resistant varieties. In this study, apoplastic ascorbate oxidase (AO) was identified as an interactor with CMV MP by screening a yeast two-hybrid library. AO is a cell wall localized enzyme, and is encoded by a multigene family with four genes each in *Cucumis melo* [28, 29] and *C*. *sativus* (cucumber.genomics.org.cn), and three genes in *A*. *thaliana* [30]. However, it is represented by single gene in tobacco, tomato and capsicum [31–33]. AO is known to control cell growth [34], cell elongation [35], auxin levels [36] and oxygen concentrations, with its role in cell wall rearrangement [37, 38]. Higher AO activity leads to increased sensitivity to ozone [39], induced stomatal closure by enhancing dehydroascorbate (DHA) levels [40] and showed a positive influence on plant growth [41]. However, lower AO activity enhanced salt tolerance [30]. Therefore, in this study, an attempt was made to identify host factors associating with the CMV MP. MP-AO association was confirmed by *in planta* methods: *in vivo* pull down and bimolecular fluorescent complementation (BiFC). Further, functional validation of the gene revealed its role in virus infection and in plant development. This study adds to the information on host factors playing important roles during viral spread at the early stages of infection.

## Materials and Methods

### Yeast two hybrid screening

The CMV MP coding region (840 bp) was amplified from the RNA3 segment of CMV isolate SG [Accession number HE583224], (a previously characterized subgroup II CMV isolated in our lab from cucumber) [42] and cloned into the pGBKT7 vector (Clontech, TakaraBio, Japan) to yield MP-pGBKT7 as bait (for yeast two-hybrid screening), which was confirmed by sequencing. Firstly, the background expression of the *HIS3* gene was suppressed by adding 3-amino-1, 2, 4-trizole (3-AT), a competitive inhibitor of the reporter gene *HIS3*, in yeast *Saccharomyces cerevisiae* strain AH109. The concentration of 3-AT suppressing background growth was selected for the screening of true interactions. Subsequently, auto activation by the MP-bait, i.e., activation of the reporter gene in the absence of any prey partner, was checked on synthetic dropout (SD) media plates SD/-LTH (lacking leucine, tryptophan and histidine) or on SD/-LTHA (lacking leucine, tryptophan, histidine, and adenine).

For cDNA library preparation, total RNA was extracted using the guanidine isothiocyanate method [43] from the pooled samples of mock-inoculated as well as CMV-inoculated *C*. *sativus* plants cv. Summer Green (SG). The virus was transferred through mechanical inoculation of pure culture of CMV-SG, which was maintained on *N*. *tabacum* cv. Samsun, and all the plants were kept in insect proof glasshouse conditions at 24±2°C. Leaf samples were collected at different time intervals: 1, 6, 12, 24, 36, 48 and 72 h at the 2–3 leaf stage and from these samples Poly (A)^+^ RNA (~500 ng) was isolated using the mTRAP kit (Active motif, USA). The RNA was used for cDNA synthesis using SMART^®^ technology of the Matchmaker library construction and screening kit (Clontech, TakaraBio, Japan). This was followed by generation of double-stranded cDNA utilizing the high fidelity 50×Advantage 2 polymerase mix (Clontech, TakaraBio, Japan). A GAL4 AD fusion library was produced by the co-transformation method in AH109. The screening was done on minimal selection media plates SD/-LTH or SD/-LTHA along with positive (pGADT7-RecT+ pGBKT7-53) and negative controls (pGADT7-RecT+ pGBKT7-Lam) for 7–10 days at 30°C. Further confirmation of positive interactions was carried out on SD/-LTHA containing 4mM AT and by β-X-gal assay.

Positive transformants were analyzed by colony PCR. The amplified products were sequenced and data was analyzed through Blast analysis of the cucumber genome database (http://cucumber.genomics.org.cn/). The partial sequences obtained from the library were taken as references to design primers for the full-length genes.

### Interaction analysis of MP with cucumber AO in yeast

**Fig 1:**
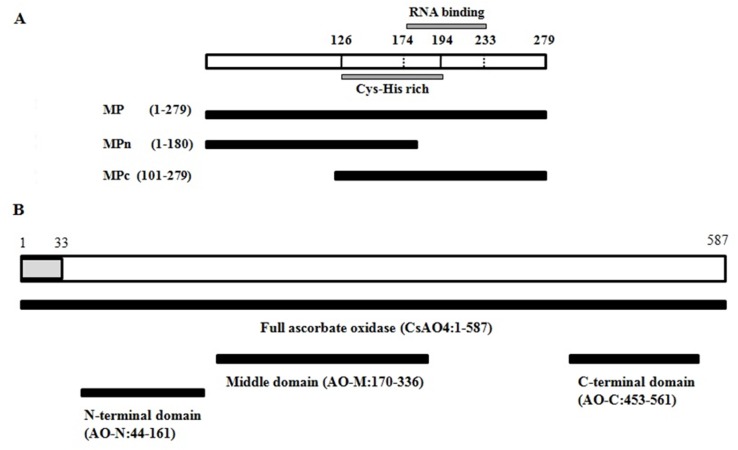
Construct preparation for yeast two hybrid. A) Diagram of CMV MP showing cysteine and histidine (Cys-His) rich region (126–194 aa); and RNA binding regions (174–233 aa). N-terminal region (MPn: 1–180 aa); C-terminal region (MPc: 101–279 aa) of MP; and complete MP (1–279 aa) used to prepare constructs in the pGBKT7 vector for yeast two-hybrid screening. B) Diagram of CsAO4 describing the location of the signal peptide, and of domains selected from three regions: Full AO (FAO: 1-578aa); N-terminal AO region (AO-N: 44-161aa); C-terminal AO region (AO-C: 170-336aa); middle AO region (AO-M: 453-561aa); and the signal peptide (1–33 aa) highlighted in grey color.

Based on the cDNA library clones sequence information, full length *AO* was amplified from *C*. *sativus* using high fidelity Advantage 2 Polymerase mix (Clontech, TakaraBio, Japan). Percent identity index of *AO* from different plants (the nucleotide sequences were taken from the NCBI database) was carried out using Bioedit version 7.1.9 software [44]. Cucumber *AO* (*CsAO4*) was cloned in the pGADT7 vector at restriction sites *Eco*RI and *Sac*I (*CsAO4*-pGADT7). Further, to identify the region responsible for interaction between two proteins, constructs were prepared from different regions of the MP region (N terminal: MPn-pGBKT7 and C terminal: MPc-pGBKT7); and AO (N-terminal: AO-N-pGADT7, Middle: AO-M-pGADT7 and C-terminal: AO-C-pGADT7) (Fig 1). Interaction between transformants was checked by co-transformation into AH109. The transformants were first selected on SD/-LT and then transferred to SD/-LTHA (4mM AT) plates. To check the specificity of interaction between CsAO4 and MP, *CsAO4*-pGADT7 was also transformed with other CMV proteins as baits: the RdRp region (2a), the 2b protein, the methyl transferase and the helicase regions (1a) (amplified from cDNA clones of CMV-SG genomic RNA). Empty bait vector and lamin C were used as negative controls.

### Structure and protein interaction prediction

The amino acid sequences of MP (HE583224) of CMV and CsAO4 (FR750377) were obtained from the GenBank (http://www.ncbi.nlm.nih.gov/genbank/). A structural model of CsAO4 was generated using the Swiss-model workspace [45], a web-based server for comparative protein modelling. The structure of ascorbate oxidase from *Cucurbita pepo* (PDB ID: 1AOZ) was used as a template for modelling. In the absence of the CMV MP structure, its *ab initio* modelling was done using i-tasser (http://zhanglab.ccmb.med.umich.edu/I-TASSER/). The quality of the 3D-model of MP was evaluated using PROCHECK tool at SAVS server (http://nihserver.mbi.ucla.edu/SAVES/). Docking was performed using the HexServer (http://hexserver.loria.fr.). CsAO4 and MP were uploaded as inputs into Hex and treated as receptor and ligand, respectively. Molecular interactions between proteins were predicted using Ligplot+ v.1.4.3 software [46].

### BiFC and *in vivo* pull down

CMV MP and *CsAO4* were cloned into pSPYNE173 and pSPYCE (MR) [47]; respectively to produce fusion with N- and C-terminal of yellow fluorescent protein (YFP) (vectors were kind gift from Prof Jorg Kudla, Germany). These binary plasmids were then transformed into *Agrobacterium tumefaciens* strain GV3101 by the freeze thaw method. Transient expression of fusion proteins was done by agroinfiltration, and cells were resuspended in agro-infiltration medium (10 mM MES, pH 5.6, 10 mM MgCl_2_, and 150 mM acetosyringone) along with cells expressing the p19 suppressor at an O.D. of 0.5:0.5:0.3. The infiltration was done on 5–6 week-old *N*. *benthamiana* leaves and the underside of infiltrated leaves were analyzed by confocal microscopy (LSM 510 Meta, Carl Zeiss, Germany) after 48 h. YFP signal was detected with an excitation at 514 nm and emission at 530–600 nm. The protein of interest along with empty vector was taken as a negative control.

For the *in vitro* pull down, protein was extracted from the infiltrated *N*. *benthamiana* leaves in extraction buffer: 50 mM Tris/HCl, pH 7.8, 0.3 M mannitol, 1 mM EDTA, 2 mM MgCl_2_ and 0.05% (w/v) cysteine [48]. Leaf tissue was homogenized in ice cold extraction buffer; centrifuged at high speed and the supernatant was collected. Protein complexes were captured with anti-HA antibody (Clontech, TakaraBio, Japan) (~1 μg) by incubation at 4°C on a rocker for 4 h. These immunocomplexes were precipitated with sure beads protein G magnetic beads (Bio-Rad, USA) overnight at 4°C on a rocker. The beads were subsequently washed four times with PBST and the immunoprecipitates eluted from the beads by boiling in 2x sample loading buffer. The eluted protein complexes were separated by 10% SDS-PAGE, transferred to a PVDF membrane and western blotted with anti-myc antibody (Clontech, TakaraBio, Japan) at a 1:4000 dilution.

### Overexpression of *CsAO4* in Arabidopsis

For overexpression in *A*. *thaliana*, constructs were prepared in the vector pCAMBIA1302 (*CsAO4*- pCAMBIA1302) and transformed into *Agrobacterium* strain GV3101. Wild type (WT) plants of Arabidopsis ecotype Columbia were grown on soil mixture of vermiculite: perlite: cocopeat (1:1:1) in the greenhouse under conditions of 16 h light and 8 h dark cycle at 20±2°C. Arabidopsis plants were transformed by the vacuum infiltration method [49]. T0 seeds were collected and grown on Murashige and Skoog (MS) medium [50] with 20 μg/mL hygromycin plates. Selected plants showing resistance to hygromycin were transferred to pots and checked for the T-DNA integration with primers for the hygromycin gene region (S1 Table). Further, seeds collected from positive plants were subjected to the same selection up to T3 generation and transgene expression was checked in WT and various transgenic lines (T3) through semi-quantitative PCR with *CsAO4* specific primers (S1 Table).

Two transgenic lines (T1 and T2) were selected to determine the effect of *CsAO4* overexpression on virus accumulation. WT and transgenic lines of Arabidopsis were mechanically inoculated with CMV-SG and leaf samples (upper non-inoculated systemic leaves) were collected at various time intervals (3, 5 and 8 dpi). Each replicate contained three plants and two leaves were taken from each plant. The CP accumulation was checked through quantitative real time polymerase chain reaction (qRT-PCR) and repeated thrice.

### Transient silencing of AO in *N*. *benthamiana* by virus induced gene silencing (VIGS) vectors

For VIGS, pBINTra6 (for TRV RNA1 [51]) and pTRV2 (for cloning host genes [52]) were used. Constructs of *N*. *benthamiana* AO, *NbAO* (Towards the 5’ region: *NbΔAO* = 735 bp) and *CsAO4* (full length, *CsFAO* = 1761 bp; and covering middle and 3’ region, *CsΔAO* = 770 bp) were prepared in the pTRV2 vector and selected regions were checked for off-target hits using pssRNAit (http://plantgrn.noble.org/pssRNAit/) and VIGS tool [53]. The regions selected for preparation of VIGS constructs were from the best identified hits to target host AO mRNA using VIGS tool [53]. The schematic representation of selected regions in cucumber and nicotiana is as given in S7 Fig. *Agrobacterium* strain GV3101 transformed with pTRV1, pTRV2, *PDS*-pTRV2, *NbΔAO*-pTRV2, *CsFAO*-pTRV2, and *CsΔAO*-pTRV2 were grown on LB agar plates supplemented with kanamycin and rifampicin (50 μg/mL each). VIGS infiltration was done on 3–4 week-old *N*. *benthamiana* plants as described earlier [54]. For effective viral spread, virus inoculated and mock controls were maintained in a growth chamber with a light period of 16 h and dark period of 8 h at 23 ± 2°C.

RT-PCR for virus confirmation: Semi-quantitative RT-PCR was done to determine the presence of TRV RNA in the inoculated plants. Total RNA was isolated from upper newly developed leaves above agro-infiltrated ones and cDNA was synthesized from 1 μg total RNA using random hexamer primers (100 ng) and PCR was done for the detection of TRV RNA1. *NbActin* gene was used for cDNA normalization in RT-PCR.

Confirmation of *AO* gene transcript levels by RT-PCR: Total RNA was isolated from upper non-inoculated leaves using an RNA extraction kit (Macherey Nagel, Germany). The transcript levels of *NbAO* in silenced plants were compared with mock controls by RT-PCR using *NbAO* full gene primers and not by VIGS construct region specific primers (S1 Table) to avoid amplification from infiltrated DNA construct.

After 11 days of transient silencing, upper leaves of VIGS silenced plants were challenge inoculated with CMV-SG. Upper non- inoculated leaves were collected to check the presence of virus at various time points. The experiment was repeated three times and each replicate contained samples from three plants.

### Expression analysis by qRT-PCR

To determine the expression level of *CsAO4* in response to CMV infection, CMV-SG was mechanically inoculated to the cotyledonary leaves of *C*. *sativus* cv. Summer Green. Total RNA was extracted from the inoculated leaves at various time points (6, 12, 24, 36, 48, 72, 96, 144 and 168 h) using Plant RNA extraction kit (Macherey Nagel, Germany). Quality and integrity was assessed by agarose gel electrophoresis and quantification was done by nanodrop (Nanodrop 2000, Thermo Scientific, USA). First strand cDNA synthesis reaction contained 1 μg total RNA (DNaseI treated), oligo dT (200 ng) and M-MLV Reverse Transcriptase (Affymetrix USB, USA). Primers were designed using Primer3 software (http://primer3.ut.ee/) and checked for the specific amplification, sequence of which was confirmed. qRT-PCR was performed with a DyNAmo Flash SYBR Green qPCR Kit (Thermo Scientific, USA) on StepOnePlus^TM^ real time PCR system (Applied Biosystems, USA). The cycling program was a three-step cycling method: 94°C for 5 min; 40 cycles of 94°C for 30 s, 58°C for 30 s and 72°C for 30 s. All reactions were performed in triplicate and no template controls were included for each primer pair. Melt curve analysis was performed on technical replicates to ensure that the qRT-PCR products include no primer dimers or multiple products. Relative gene expressions of *CsAO4* were calculated using the 2^-ΔΔCt^ method [55]. Cucumber *Actin* was used as internal control to normalize the amount of *AO* mRNA for each sample. Gene expression of each time point was quantified relative to the mock inoculated control for that particular time point. In all replicates, each sample comprised of leaves from three plants.

Relative virus titer was measured in terms of CP accumulation in CMV inoculated *CsAO4* overexpressing lines of Arabidopsis/VIGS silenced plants with *NbΔAO*-pTRV2 by qRT-PCR. WT Arabidopsis/empty vector (pTRV vectors) infiltrated *N*. *benthamiana* plants were taken as controls in respective experiments. Reaction and temperature conditions for qRT-PCR were same as mentioned above. 18S rRNA was taken as endogenous reference gene in virus titer experiments. Relative CP accumulation was calculated in comparison to 3 dpi CMV-infected vector control/WT plants in respective experiments.

## Results

### Identification of plant host factors interacting with CMV-MP

Intracellular movement of the virus depends on interaction of MP with various host factors. Considering that, *in vivo* generated cucumber expression library in pGADT7 was screened with MP as bait having co-transformation efficiency of approximately 2.2×10^5^ cfu/μg. Before screening, auto-activation potential of the bait was checked and leaky expression of *HIS3* gene in AH109 strain was inhibited by 4mM 3-AT in SD/-TH media plates. A number of colonies showing good growth on SD/-LTHA/+4mM 3-AT were selected and prey plasmids were rescued from these colonies. On sequencing, the cDNA fragments from three positive interactors showed high degree of similarity (95–98%) with L-*Ascorbate oxidase* (*AO*) of *C*. *sativus*, J04494 in the NCBI database (http://www.ncbi.nlm.nih.gov/) and with Csa019416 in the cucumber genome database (http://cucumber.genomics.org.cn/). AO is a glycoprotein, member of multi-copper oxidase family and localized in the cell wall. The enzyme oxidizes ascorbic acid using molecular oxygen to DHA via an intermediate monodehydroascorbate. The enzyme is a homodimer and each of its subunit contains three distinct cupredoxin domains. Domain 2 and 3 are involved in monomeric interaction through side chains of β-turns or side coils. Each monomer contains 14 tryptophan, 23 tyrosine residues and four copper ions [56–58].

The complete coding region of cucumber *AO* was amplified through RT-PCR and the sequence was submitted to the EMBL database (accession number FR750377). The full length *AO* is 1764 bp; encoding a 587-amino acid (aa) protein consisting of a signal peptide of 33 aa at the N-terminal region and a mature peptide of 554 aa (Fig 1B). Its ClustalW alignment showed greater similarity with the *AO4* homolog of melon (S1 Fig). The gene showed 94.1% nucleotide sequence identity with melon *AO4* (AF233594); 79.8% identity with *AO1* (AF233593); 53.1% with *AO2* (XM_008442414); and 66.8% with *AO3* (Y10226). It was found to show 64.3% similarity with *N*. *benthamiana AO* (HG938363) (S2 Fig). From here onwards, *AO* from cucumber and *N*. *benthamiana* are therefore described as *CsAO4* and *NbAO*, respectively.

### Interaction of MP with AO domains

**Fig 2:**
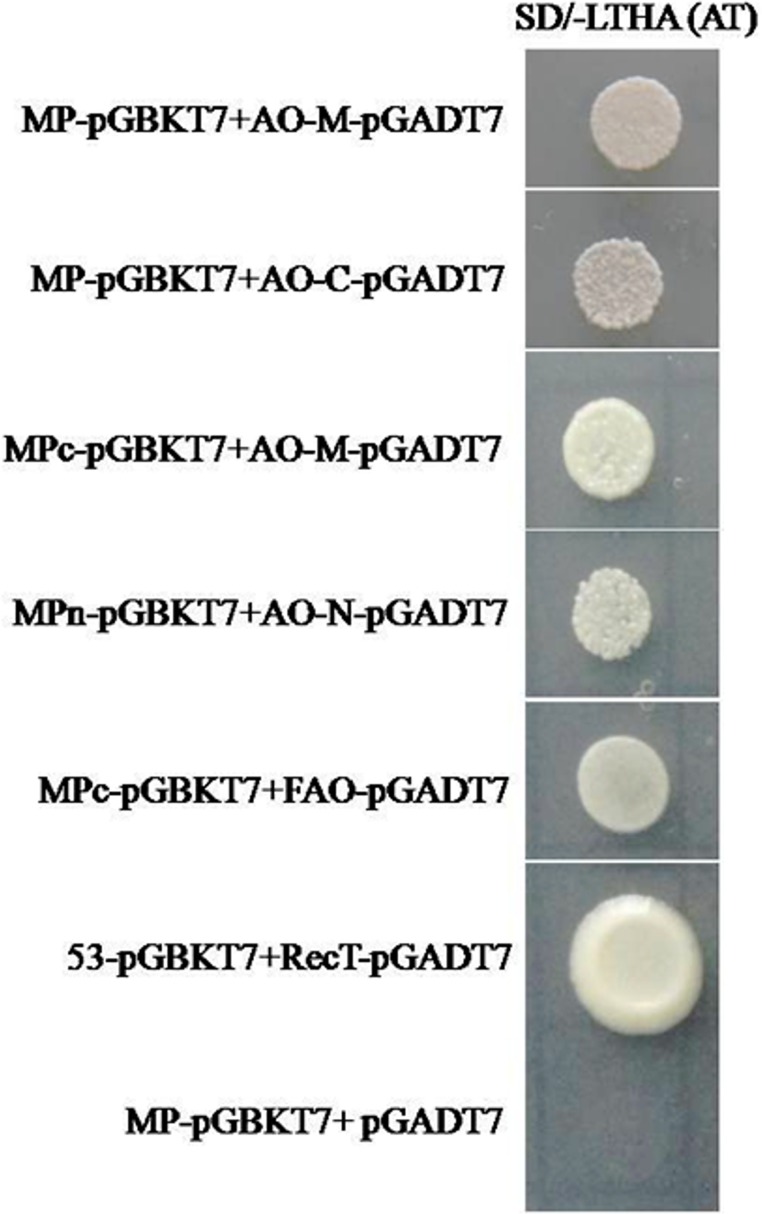
GAL4 Yeast two hybrid assay. Yeast transformants spotted on selection medium (SD/-LTHA/+4mM AT) showing interaction of full length AO and its domains with MP domains along with positive (SV40 Large T antigen and p53) and negative (MP-pGBKT7 and empty pGADT7 vector) controls. Abbreviations used in the figure: MPc (C-terminal region of MP); FAO (Full length CsAO4); MPn (N-terminal region of MP); AO-N (N-terminal, cupredoxin domain 1 region of CsAO4); AO-M (Middle region, cupredoxin domain 2 region of CsAO4) and AO-C (C-terminal, cupredoxin domain 3 region of CsAO4).

Interactors from the cDNA library screening covered the 3’ region of cupredoxin domain1 (AO-N) and complete domain 2 (AO-M) of CsAO4, and showed growth on selection plates (SD/-LTHA/+4mM AT). However, full length CsAO4-pGADT7 did not show growth on co-transformation with MP-pGBKT7. The larger size or aggregate formation may prevent proper folding of the protein, restricting their interaction. Therefore, the interaction was dissected out at the region/domain level. The MP was divided randomly (Fig 1A) into N-terminal (MPn-pGBKT7) and C-terminal regions (MPc-pGBKT7), while CsAO4 was divided into different regions for the cupredoxin domains (AO-N-pGADT7, AO-M-pGADT7 and AO-C-pGADT7) (Fig 1B). MPn-pGBKT7 consisted of 180 aa (1–180); MPc-pGBKT7 of 179 aa (101–279) and both constructs covered the common region of 80 aa (Fig 1A). During this screening, we found that co-transformants MP-pGBKT7/AO-M-pGADT7, MP-pGBKT7/AO-C-pGADT7, MPn-pGBKT7/AO-N-pGADT7, MPc-pGBKT7/AO-M-pGADT7, and MPc-pGBKT7/CsAO4-pGADT7 showed growth on -LTHA (4mM AT) (Fig 2) and was also positive during β-galactosidase assay. The dilution assay was also performed for yeast transformants to determine the strength of interactions (S3 Fig).

Schematic representation of protein-ligand interaction using the LigPlot+ program is shown in S4 Fig. The diagram represents the hydrogen and hydrophobic interactions between CsAO4 (chain A) and MP (chain B). MP is shown interacting with the Glu496 and Thr286 residues of AO through hydrogen bonding, but with Asn360, Phe284, Pro284, Gly497, Asp354, Arg357, Gly495, Phe363, Arg364, Lys507, Tyr494, Trp526, Glu503, Asp304, Tyr500, Ile438, Asn439, Tyr524 and Asp356 through hydrophobic interactions. The CsAO4 amino acids involved in interactions were from cupredoxin domains 2 and 3.

### CMV-MP interacts with CsAO4 *in planta*

**Fig 3:**
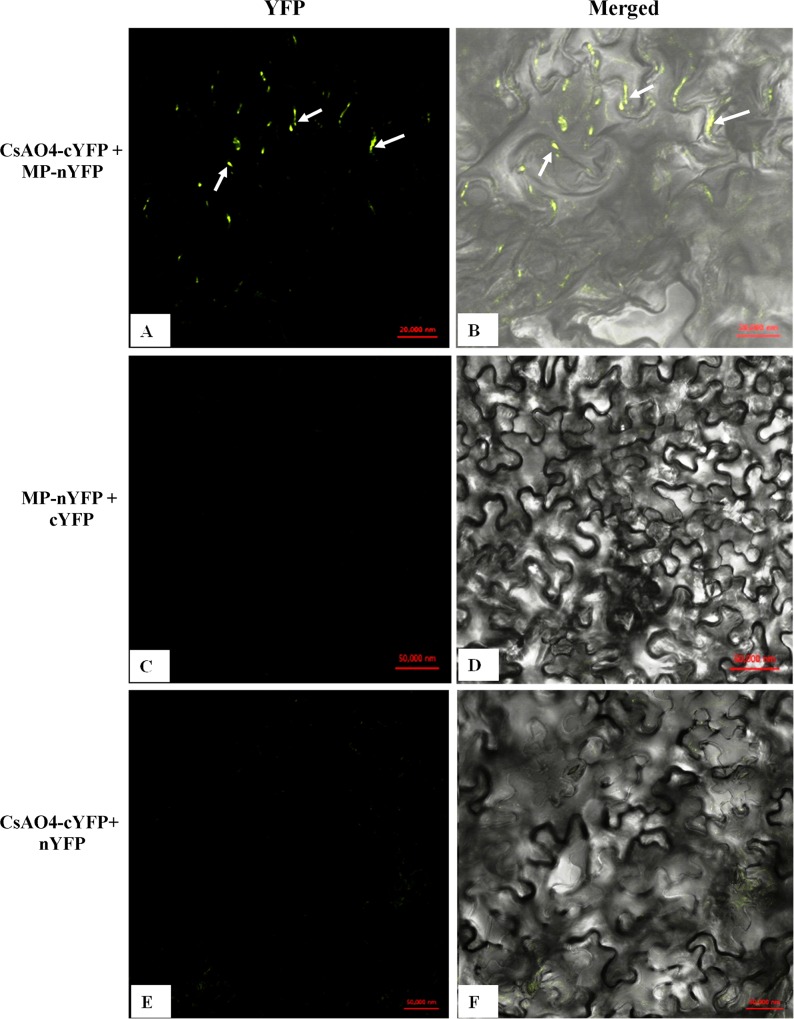
*In planta* interaction between CsAO4 and CMV MP by BiFC. The lower surface of *N*. *benthamiana* leaves was observed under the confocal microscope for fluorescence from YFP: A) and B) CsAO4-cYFP and MP-nYFP. C) and D) MP-nYFP and cYFP. E) and F) CsAO4-cYFP and nYFP. YFP reconstitution observed in A and B showed punctate sites around the cell wall periphery. Confocal images were merged with bright field images. Fluorescence was detected 48 h post agroinfiltration. Scale bars are shown in the figures.

*In-vivo* protein-protein interaction between CMV-MP and CsAO4 was confirmed by BiFC assay in *N*. *benthamiana*. CsAO4 was fused with the C-terminal half of YFP (CsAO4-cYFP); and MP was fused with the N-terminal half of YFP (MP-nYFP). Proteins were infiltrated along with the silencing suppressor p19 and lower epidermal cells were observed for reconstituted YFP signals. Fluorescence was detected only in presence of CsAO4-cYFP and MP-nYFP (Fig 3A and 3B). Fluorescence was observed around the cell wall periphery as punctate spots. No distinct fluorescent signals were found except some background fluorescence in control experiments, when expressing MP-nYFP/cYFP (Fig 3C and 3D) and CsAO4-cYFP/nYFP only (Fig 3E and 3F).

**Fig 4:**
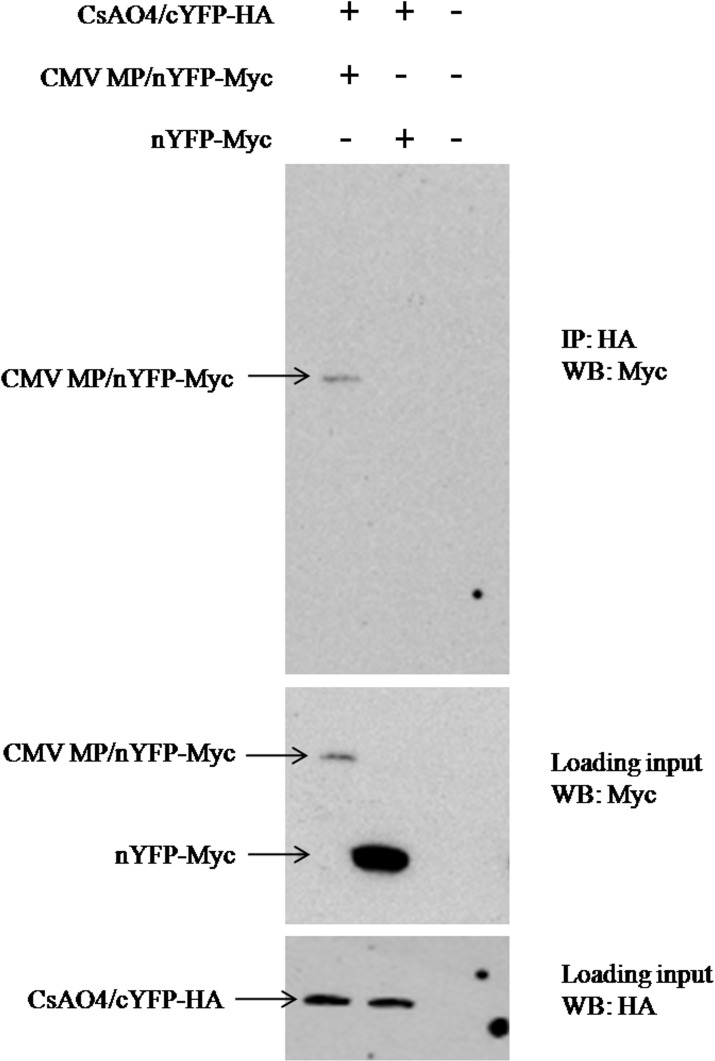
*In vivo* pull down assay showing interaction between MP and CsAO4. Protein complexes were immuno-precipitated using anti-HA antibody (raised in rabbit) and analyzed by SDS-PAGE. Proteins were transferred to PVDF membranes and probed with anti-c-Myc antibody. Abbreviations: MP-nYFP (complete MP in pSPYNE173 vector), CsAO4-cYFP (complete AO in pSPYCE (MR) vector), nYFP (empty pSPYNE173 vector) and cYFP (empty pSPYCE (MR) vector).

For the *in vivo* pull down assay, protein was extracted from leaves transiently expressing CMV MP-nYFP and CsAO4-cYFP in BiFC vectors. Proteins were extracted using a low ionic strength buffer suitable for extraction of cell wall proteins [48], as MP and AO were reported to be localized around the cell wall (PD/apoplast). Therefore, the low ionic strength buffer worked well in protein isolation and subsequent detection by epitope specific antibodies (Anti-c-Myc/HA) in western blotting. Protein complexes were captured using Anti-HA tag antibodies (for CsAO4 fusion) and precipitated using protein G magnetic beads. Subsequent detection was done with c-myc antibodies (for CMV MP fusion) which lead to the detection of bands corresponding to the size of the MP-nYFP fusion on the membrane (Fig 4). The results of this assay supported interaction between CsAO4 and MP in plant cells.

### Differential response of CsAO4 in cucumber during CMV infection

**Fig 5:**
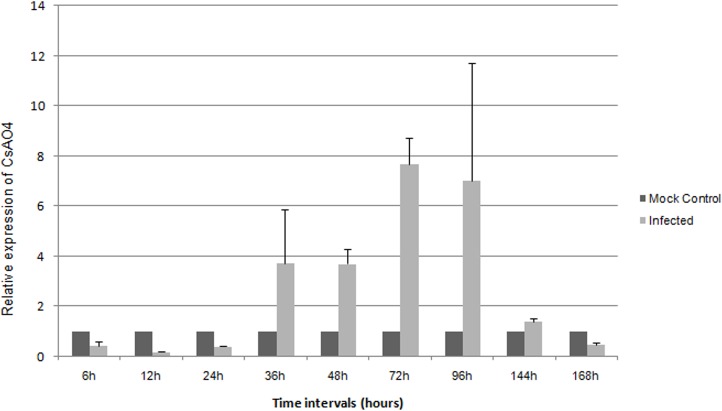
Relative *AO* expression levels in *C*. *sativus* cv. Summer Green at different time points during CMV infection. Buffer inoculated plants served as control samples for each time interval. Relative expression levels were determined at various time points (6, 12, 24, 36, 48, 72, 96, 144 and 168 h) in comparison to mock inoculated plants at the same time points. The cucumber *Actin* gene was used to normalize all data and error bars illustrated the standard deviation about the mean for three independent biological replicates. Relative expression was plotted as 2^-ΔΔCT^ (fold change) values. h- hours post inoculation.

To determine the effect of CMV infection on *CsAO4* expression, qRT-PCR analysis was carried out. CMV-SG developed visible symptoms in 3–4 days on cucumber leaves. Leaf samples of cucumber were collected at different time points up to 168 h from inoculated and mock treated plants. In melon, *AO4* expression was reported to be affected by wounding stress [29]. Considering this, gene expression in infected plants was determined in comparison to mock control plants of the same time points. After infection, *AO* transcript levels were repressed at early time points (up to 24 h) followed by upregulation of 3.7 fold and reached a maximum of about 7.6 fold at 72 h in comparison to the mock control. Subsequently, there was gradual decline in transcript level but expression remained higher in comparison to mock plants (Fig 5).

### Effect of *CsAO4* overexpression on CMV accumulation

**Fig 6:**
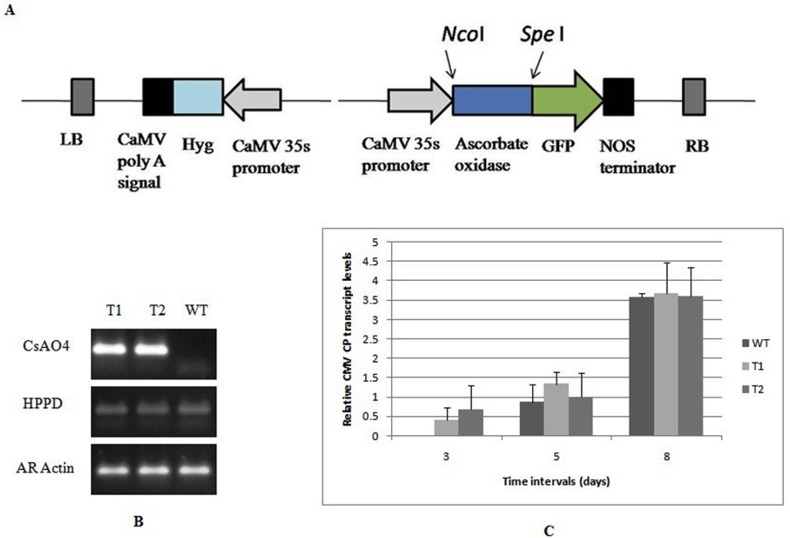
Overexpression of *CsAO4* in *A*. *thaliana*; Analysis of transgene integration and effect of overexpression on CMV infection. A) Diagrammatic representation of *CsAO4* constructs in pCAMBIA1302 vector as GFP fusion product. CaMV 35S promoter: *Cauliflower mosaic virus* 35S promoter, GFP: Green fluorescent protein, Hyg: Hygromycin, LB: left border, RB: right border. B) Validation of *CsAO4* transgene expression in Arabidopsis transgenic lines and WT by RT-PCR. Arabidopsis actin (AR *Actin*) was used as an internal control, *HPPD* (*4-Hydroxy phenyl pyruvate dioxygenase*) is a single copy gene in Arabidopsis and *CsAO4* is the cucumber *Ascorbate oxidase 4*. C) Relative CMV CP accumulation in *CsAO4* overexpressing Arabidopsis lines and WT plants. Relative CP accumulation in infected transgenic plants was calculated relative to CMV infected WT plants at 3 dpi. The 18S RNA gene was used to normalize all data and error bars represent the standard deviation about the mean for three independent biological replicates. Relative expression was plotted as Log2 values; dpi—days post inoculation.

To investigate the effect of *CsAO4* overexpression, transgenic constitutively-expressing Arabidopsis lines were generated using *CsAO4*-pCAMBIA1302 (Fig 6). These lines did not exhibit any phenotypic variation in comparison to WT except for early flowering (S5 Fig). Two T3-generation transgenic lines (T1 and T2) as well as WT plants were checked for viral CP RNA accumulation at 3, 5 and 8 dpi. Transgenic lines showed only mild increase in CP RNA accumulation in comparison to wild type control plants at 3 and 5 dpi. CP RNA accumulation reached almost similar levels at 8 dpi, and no significant difference in expression was seen at this stage (Fig 6C). These results showed that overexpression of *AO* did not affect virus accumulation in arabidopsis significantly during early infection. However, anthocyanin pigmentation was observed on leaves of infected transgenic plants and not on infected WT plants (S6 Fig).

### *AO* silencing by VIGS reduced CMV accumulation in *N*. *benthamiana* at early stages

**Fig 7:**
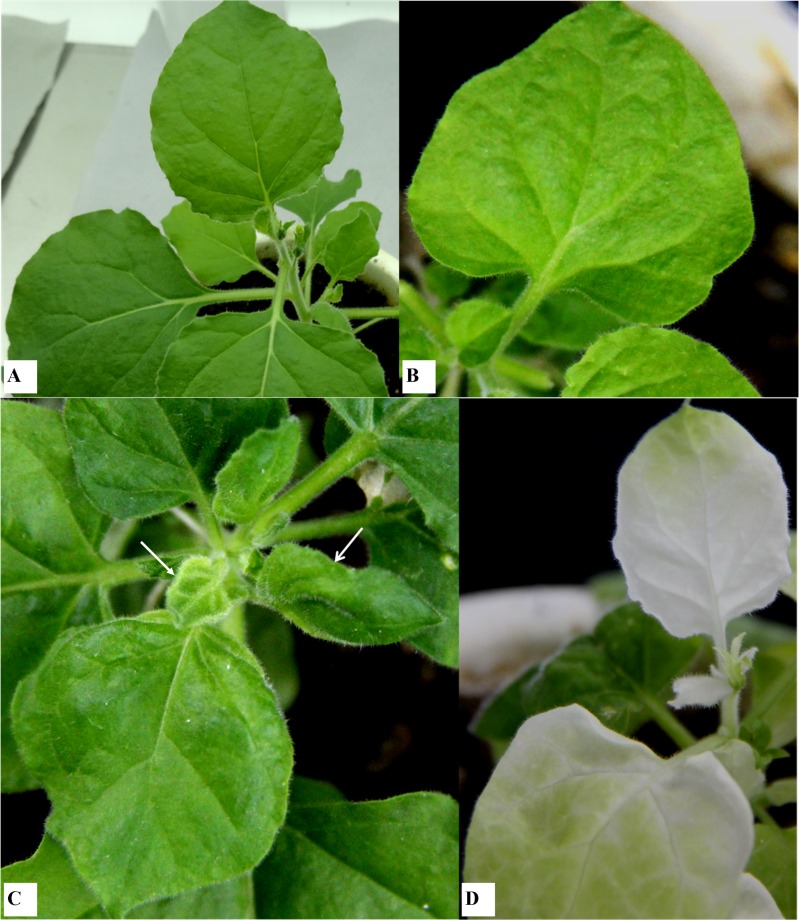
Effect of *NbAO4* (VIGS) silencing on *N*. *benthamiana* two weeks post agro-infiltration. A) Mock control plant: developed no symptoms. B) Empty TRV vector: mosaic symptoms. C) Partial *N*. *benthamiana AO* (*NbΔAO*-pTRV2): caused downward leaf curling at earlier stages and then severe leaf distortion affecting leaf lamina and developing chlorosis, as indicated by arrows. D) *PDS*-pTRV2 control: newly emerging leaves showed photo bleaching effect.

**Fig 8:**
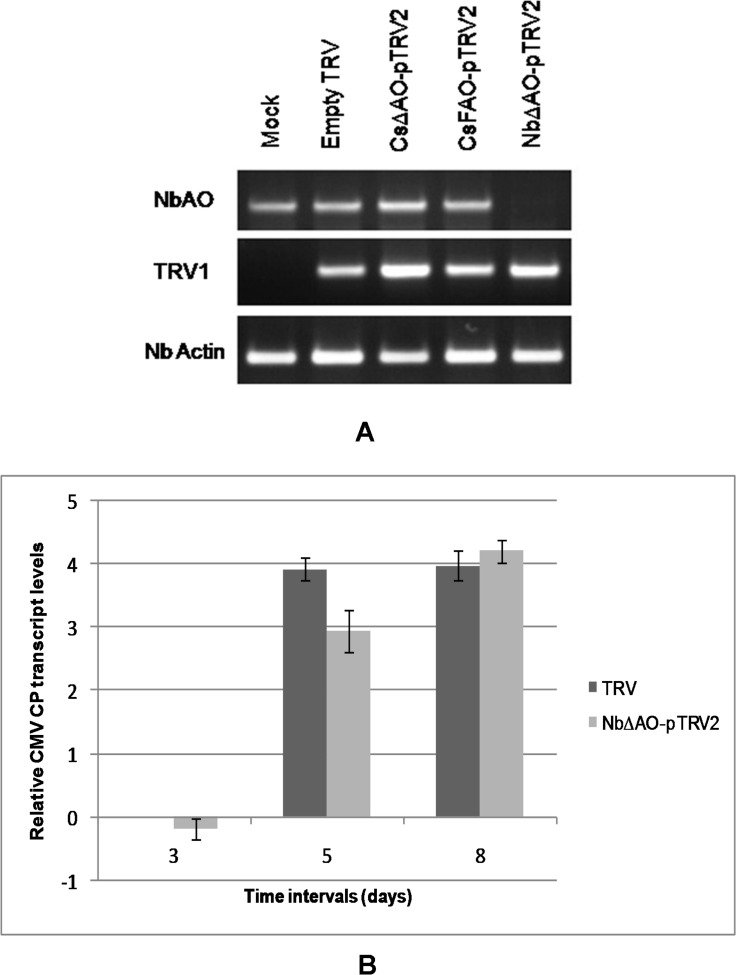
Quantification of *NbAO* and CMV CP RNA levels in silenced plants. A) Transcript levels of *NbAO* were assessed by semi-quantitative RT-PCR using complete gene primers. *NbActin* was used as an internal control and TRV infection was checked by detecting the presence of TRV RNA1. B) Relative CMV CP RNA accumulation in TRV control and *NbΔAO*-pTRV2 silenced plants. CP RNA accumulation in *AO* silenced plants was calculated relative to CMV-infected TRV control plants at 3 dpi. The 18SrRNA gene was used to normalize all data and error bars represent the standard deviation about the mean for three independent biological replicates. Relative expression was plotted as Log2 values.

To understand the effect of *AO* transient suppression on the viral spread, TRV-VIGS vector was used to suppress the *N*. *benthamiana CsAO4* gene (http://solgenomics.net/), which was 97.6% and 64.3% identical at the nucleotide level to the *N*. *tabacum* (D43624) and *C*. *sativus* (FR750377) genes, respectively (S2 Fig). Along with *NbAO* (*NbΔAO*- pTRV2), *CsAO4* (*CsFAO*-pTRV2 and *CsΔAO*-pTRV2) constructs were used for transient silencing of *AO* (S7 Fig). Results showed that low sequence identity can also affect silencing. After 8 days after agro-infiltration, the phenotypic effect of silencing *phytoene desaturase* started appearing on positive control plants and similarly, symptoms of silencing *NbAO* appeared on newly emerging leaves of silenced plants. Plants infected by *NbΔAO*-pTRV2 at first developed necrosis around veins and mosaic appearance in leaves above the inoculated ones, but later, exhibited marked effect on leaf development, like curling of leaves and deformation of leaf lamina (Fig 7). Similarly, plants infected with the *CsAO4* silencing constructs (*CsFAO*-pTRV2 and *CsΔAO*-pTRV2) also showed symptoms like necrosis around veins in leaves above inoculated ones, and later, leaf deformation along with chlorotic patches (S8 Fig). TRV RNAs were detected in non-inoculated upper leaves of all inoculated plants through RT-PCR, showing its systemic movement in the plant. The expression of *NbAO* was reduced significantly in *NbΔAO*-pTRV2 silenced plants in comparison to mock inoculated plants indicating effective silencing (Fig 8). As single copy of the *AO* gene was reported in tobacco, silencing of the homologous *N*. *benthamiana* gene was considered specific. But no major effect was observed on transcript levels of *NbAO* in *CsFAO*- and *CsΔAO*-silenced plants indicating poor efficiency regardless of observed symptoms (Fig 8).

Challenge inoculation with CMV-SG was done on the upper leaves of *NbΔAO*-silenced and TRV control plants 11 days after TRV infiltration. Relative virus accumulation was then analyzed in these silenced plants at various time points post inoculation through real time-PCR in terms of CP RNA accumulation, using TRV inoculated plants as controls. At 5 dpi, less CP RNA accumulation was seen in *NbΔAO*-silenced plants as compared to TRV controls. This low accumulation could be due to hindrance to viral spread caused by *AO* gene silencing. At 8 dpi, CP RNA levels in silenced plants also reached to the level of control ones (Fig 8). These results suggested a role for AO during initial infection events.

## Discussion

Recruiting host factors on entering the cells helps plant viruses to spread during infection. CMV MP was known to target PD and increase their SEL by deteriorating actin filaments [7, 59], but little is known about host proteins association with the MP. During yeast two-hybrid screening, CMV MP was found to interact with the cupredoxin domain2 of CsAO4, a glycoprotein present in the apoplast of the cell wall. However, we were not able to find interaction between the full length MP and CsAO4 in yeast cells. Protein interaction in any system is dependent upon appropriate folding of the expressed protein. Further, many proteins are modular in nature and contain specific domains essential for protein-protein interactions. During library screening, random fragments of prey proteins exposing these specific domains showed interaction with bait protein [60]. During this study also, interactions were obtained at domain level in yeast two hybrid but were not successful while using full length proteins. Therefore, failure of interaction between full length proteins may be attributed to inadequate exposure of the active residues which are essential for interaction. Other reports also described such problems of protein interactions involving large molecular weight proteins because of unstable expression of large ORFs in yeast [61]. In this study, GAL4 based yeast two hybrid system was used in which fusion proteins are targeted to the nucleus [62]. Ascorbate oxidase is an apoplastic protein and contains signal peptide for its targeting to the apoplast. The presence of signal peptide might be affecting its targeting to the nucleus when full length protein was used (along with signal peptide). Another reason for improper protein interactions may involve post translational modifications which occur differently in yeast and plants. These include phosphorylations, disulfide bridges and glycosylation [62, 63]. As ascorbate oxidase is a glycosylated protein, improper posttranslational modification may lead to interference with interaction. There were some reports where full length proteins did not show interaction in yeast. The C-terminal region of the 60K protein of *Cowpea mosaic virus* showed interaction with Arabidopsis proteins but not the full length 60K [64]. The CaMV P6 protein was also found to interact with the coiled-coil domain of CHUP1 (Chloroplast Unusual Positioning 1) protein of Arabidopsis [65]. Therefore, identification of domains involved in interaction was performed through yeast two hybrid system. Full length protein interactions were confirmed by *in vivo* pull down and BiFC assays. Association of CsAO4 and CMV MP around the cell wall periphery at punctate sites suggested its involvement in viral transport. This is in accordance with previous findings of ‘NbGAPDH A’ interaction with RCNMV MP and rubisco small subunit interaction with the MPs of ToMV and TMV [18, 66].

During CMV infection, *CsAO4* expression levels were induced in *C*. *sativus* (cv. Summer Green) only after 24 h, and showed reduction in the transcript levels after 72 h. These observations pointed towards its involvement at the host-pathogen interface. Melon *AO4* was also found to show predominant expression in vegetative tissues (shoot apices and stem) and induction post wounding and heat shock stress but no change was observed in the levels of other family members [29]. The capsicum *AO* expression level was reported to increase due to wounding stress in the beginning and subsequently declined [33].

Altered AO levels can modulate internal as well as external signal perception in the plant. AO overexpressing lines were known to affect hormone responses [67], and stomatal closure via increased DHA content [40]. It was observed that AO controlled vital function of plant development and interference led to a marked visible effect on the plant phenotype. VIGS silencing affected leaf development and growth in AO silenced plants and similar alteration in leaf morphology was also reported in AO silenced lines of tomato [68]. Its overexpression induced early flowering in transgenic lines of Arabidopsis as compared to WT plants. Earlier studies also reported similar response in overexpressing lines of *AO* in *N*. *tabacum*. Plants overexpressing AO developed flower buds earlier to WT plants; but antisense plants had delayed bud formation. Similarly, T-DNA mutants of Arabidopsis exhibiting low AO activity also had delayed flowering [30]. Anthocyanin accumulation was found in CMV-infected, *AO* overexpressing lines but not in WT plants. This finding is in accordance to earlier reports where anthocyanin accumulation was observed upon pathogen infection in Arabidopsis. However, accumulation did not affect pathogen titer, but resulted in reduced cell death response [69]. The results obtained showed host response to stress imposed by pathogen infection in *AO* overexpressing lines.

The results here showed that overexpression did not significantly affected virus titer levels at 7 dpi and found to have only mild effect on viral accumulation during early stages of infection (5 dpi). Earlier studies with transgenic *AO* overexpressing lines were also known to show enhanced susceptibility to pathogens like *Pseudomonas syringe* (virulent strain) and *Botrytis cinerea* [67, 70]. However, knockdown of *NbAO* resulted in reduced virus accumulation at an early stage of infection (5 dpi) in comparison to the TRV control plants during this study. This observation suggested that silencing of AO compromised movement of the virus in comparison to TRV control plants which lead to reduction in virus titer. In an earlier study, transient knockdown of plastocyanin (which was found to associate with CP of *Potato virus X*) in *N*. *benthamiana* reduced *Potato virus X* CP accumulation in the chloroplasts but did not completely abolish it. The possible reason was leakiness in transient gene silencing [71]. To the best of our knowledge, an effect of *AO* silencing on pathogen infection has not been reported.

Previous studies have reported the role of host proteins with signal peptides in viral movement by utilizing the ER pathway. It was found that *Tobacco mosaic virus* MP interacted with PD localized calreticulin protein, carrying a signal peptide. Overexpression of the latter affected the cell-to-cell movement of the viral MP by interfering with its targeting, probably by overloading or blockage of PD sites [16]. PME, a cell wall associated enzyme, also reduced virus accumulation and symptom severity of TMV in PME VIGS-silenced plants [72]. Another host protein, chloroplast phosphoglycerate kinase containing a transit peptide interacting with the 3’UTR of *Bamboo mosaic virus*, was shown to target viral RNA to chloroplasts and its knockdown reduced virus accumulation [73, 74].

Like plant proteins, viral proteins also contain regions which help their targeting to various cellular organelles. Studies showed that CPs of *Cucumber necrosis virus* [75] and *Lolium latent virus* [76], and the TGB3 of *Alternanthera mosaic virus* [20, 77] possess regions which function as transit peptides required for chloroplast targeting. Although CMV MP was described as carrying a signal for cell-to-cell movement [59], so far there are no reports related to transit peptides or residues on the protein necessary for its targeting to host components. TMV MP, a member of 30K superfamily, like CMV MP, was reported to associate with the ER membrane and cytoskeletal elements for viral genome transport [78–81]. Association of the CMV MP with ER membranes is not known. However, it was reported to severe actin filaments in tobacco to increase SEL of PD [7].

Localization of AO in the apoplast of the cell wall [39, 82] and of CMV MP around PD is well established [83]. Similar results were obtained during subcellular localization studies of AO and MP in onion epidermal cells (S9 Fig). BiFC results also validated this finding. It has been reported that WT or even mutant CMV MP bound viral RNA (vRNA) and these complexes appeared like beads on string and the bound protein protected vRNA segments against RNase action [26, 84]. It was hypothesized that CMV MP associates with AO near the cell wall periphery and this association might help in movement of vRNA-MP complexes to PD during AO targeting to the apoplast. *CsAO4* upregulation in cucumber during early events of infection could be a virus induced response to facilitate virus spread in the cells. Effect of *AO* overexpression and VIGS-silencing showed that it played an important role in plant development and the virus utilizes this host factor during onset of infection. As discussed earlier, initially AO silencing developed chlorosis pattern on the leaves but eventually lead to severe leaf malformation, due to which it was difficult to observe the difference in CMV symptoms in infected AO silenced and TRV control *N*. *benthamiana*. It is quite possible that observed low viral titer during early infection in AO silenced plants was due to these developmental abnormalities. In the literature, information regarding the effect of AO silencing on cellular structures is not available. However, for bacterial and fungal pathogens, it has been reported that alteration in the AO activity (overexpression) in apoplast affects its redox state which in turn affects cellular signaling cascades, calcium signaling, antioxidant defense and hormonal responses. These changes can affect plant responses to external environmental stresses including abiotic as well as biotic [39, 67, 70]. Another explanation for the functional relevance of this association is that initially overexpression of the *AO* (plant defense response) is utilized by the virus to increase the number of receptors available for viral MP in the apoplast. This, in conjunction with targeting of the MP, helps the virus to transport more of the protein to organelles. Further, as AO functions as a dimer, association with MP is likely to reduce the dimer formation, diminishing the defense response of the plant. In conclusion, this study provides the first information on association of CMV MP with a host factor containing a signal peptide.

## Supporting Information

S1 Fig Clustal W alignment of CsAO4 amino acid sequence with other melon AO homologs. Homology between Cucumber AO under study and melon AO4 was shown in rectangular boxes. Conserved regions were shaded in grey colours.
(TIF)

S2 Fig Analysis of *CsAO4* (FR750377) nucleotide sequence. (A) Phylogenetic analysis of cucumber *AO4* with other *AO* sequences taken from different plants by Neighbour-joining method in MEGA 6.0. Plants and their accession numbers used in study were mentioned in the phylogenetic tree. Bootstrap values were indicated at branches and evolutionary distance was shown below the figure; (B) Percent identity matrix of nucleotide sequences of AO from different plants. Sequence identity of *CsAO4* with other cucumber homolog’s (*CsAO1*, *CsAO2*, and *CsAO3*) was shown in red letters; and with melon homolog’s (*M AO1*, *M AO2*, *M AO3*, and *M AO4*) was shown in grey shaded bold letters. Percent identity matrix was 1calculated using Bioedit software (version 7.1.3.0). *CsAO1* (Cucumber AO1:XM_004144064), *CsAO2* (Cucumber AO2:XM_004173026), *CsAO3* (Cucumber AO3:XM_004144107), Cucumber *AO* (J04494), Pumpkin (D55677), *M AO1* (Melon AO1:AF233593), *M AO2* (Melon AO2:XM_008442414), *M AO3* (Melon AO3:Y10226), *M AO4* (Melon AO4:AF233594), Nb (*N*. *benthamiana*:HG938363), Nt (*N*. *tabaccum*:D43624), Arabidopsis [AR(1): AT5G21105, AR(2):AT4G39830, AR(3): AT5G21100], Prunus (KC152937), Tomato (AY971876), and Maize (EU973211).
(TIF)

S3 Fig Dilution assay of yeast transformants on selection medium (SD/-LTHA/+4mM AT). The figure showing strength of interaction of full length AO and its domains with MP domains as indicated by growth on selection plate along with positive and negative controls.
(TIF)

S4 Fig Interaction plot for CMV MP and CsAO4. Based on the results of LigPlot+ interaction plot was generated showing hydrogen and hydrophobic interactions between residues. Chain A and Chain B denotes CsAO4 and CMV MP respectively.
(TIF)

S5 Fig Effect of *CsAO4* overexpression on plants. Early flowering observed in AO-overexpressing transgenic lines in comparison to wild type plants. Representative pictures of T2 plants (Four lines: A1, A2, A3, A4) and WT were shown.
(TIF)

S6 Fig Effect of CMV on WT and transgenic plants. Anthocyanin pigmentation was observed in AO-overexpressing transgenic lines in comparison to wild type plants. Representative pictures of upper and lower leaves of CMV infected transgenic (two lines: A1, A2) and WT plants after two weeks were shown in the figure.
(TIF)

S7 Fig Regions of *NbAO* and *CsAO4* used to prepare to VIGS constructs. The figure showed regions of NbAO (Nb*Δ*AO) and CsAO4 (CsFAO and Cs*Δ*AO) indicated by blue bars and cupredoxin domain 1 (CuRO1), domain 2 (CuRO2) and domain 3 (CuRO3) indicated by solid black bars.
(TIF)

S8 Fig *NbAO* silencing using *CsAO4* constructs. Phenotypic effect of NbAO knockdown was observed on plants. (A-B) Full length *CsAO4*-pTRV2 (CsFAO-pTRV2): developed chlorotic patches along with necrotic areas above infiltrated leaves; (C) Partial *CsAO4*-pTRV2 (*CsΔAO*-pTRV2): caused symptoms like leaf deformation, necrotic areas, and size reduction in new emerging leaves.
(TIF)

S9 Fig Subcellular localization of CMV MP and CsAO4 using biolistic approach. pCAMBIA1302 constructs were bombarded on onion epidermal peels by biolisitc method and GFP fluorescence was observed under fluorescent microscope. (A) Empty pCAMBIA1302 vector showing free GFP localization in cytoplasm and nucleus. (B) CMV MP-pCAMBIA1302 and (C) CsAO4-pCAMBIA1302 showing localization around cell wall region.
(TIF)

S1 Table Primers used during the study. (DOCX)
