# Supplementary figures and images for: Movement Protein of Cucumber Mosaic Virus Associates with Apoplastic Ascorbate Oxidase
Source: PLoS One. 2016 Sep 26;11(9):e0163320. doi: 10.1371/journal.pone.0163320 (PMC5036820; doi:10.1371/journal.pone.0163320)

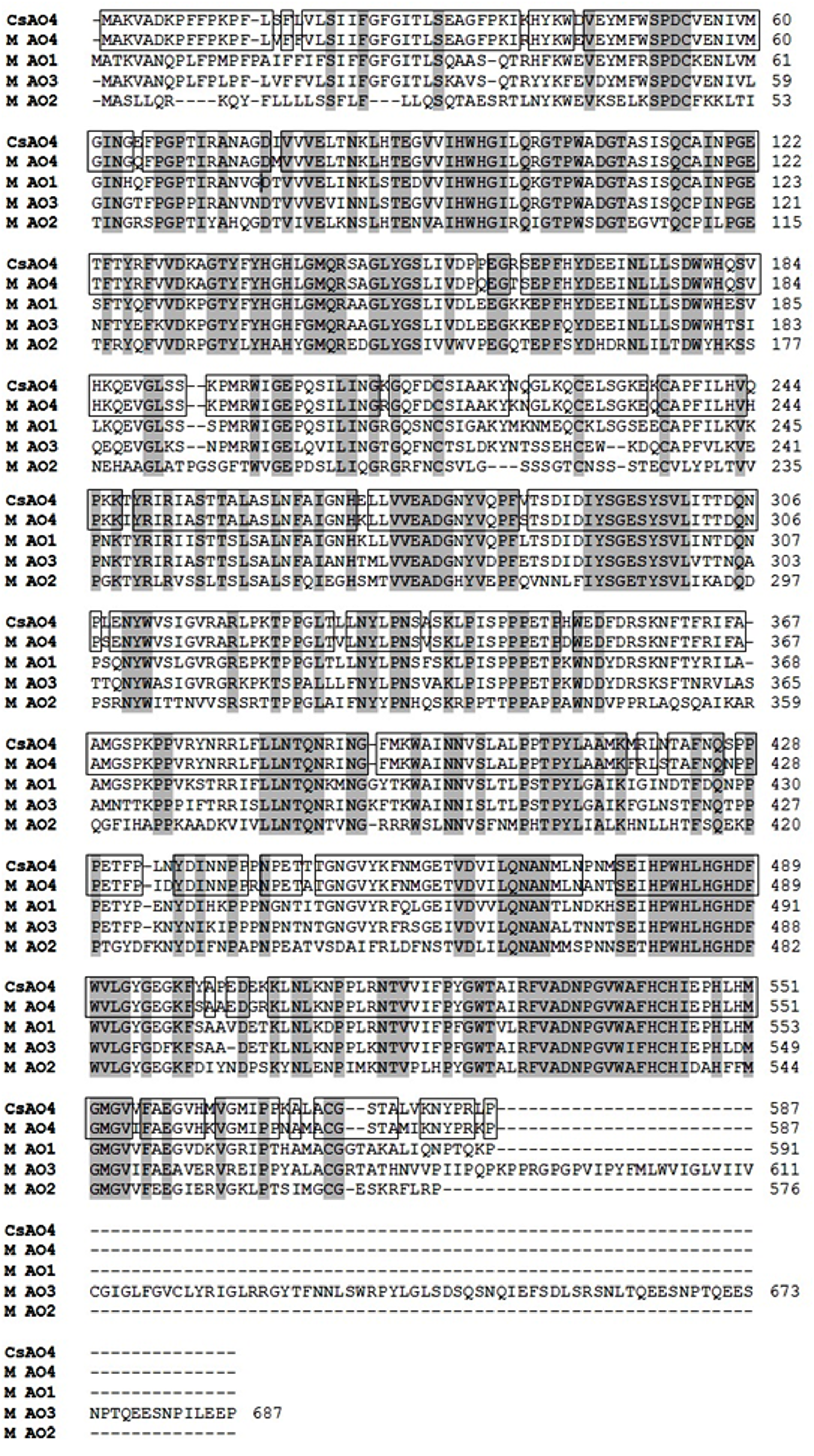

Supplement: S1 Fig — Homology between Cucumber AO under study and melon AO4 was shown in rectangular boxes. Conserved regions were shaded in grey colours. (TIF) [file pone.0163320.s001.tif]

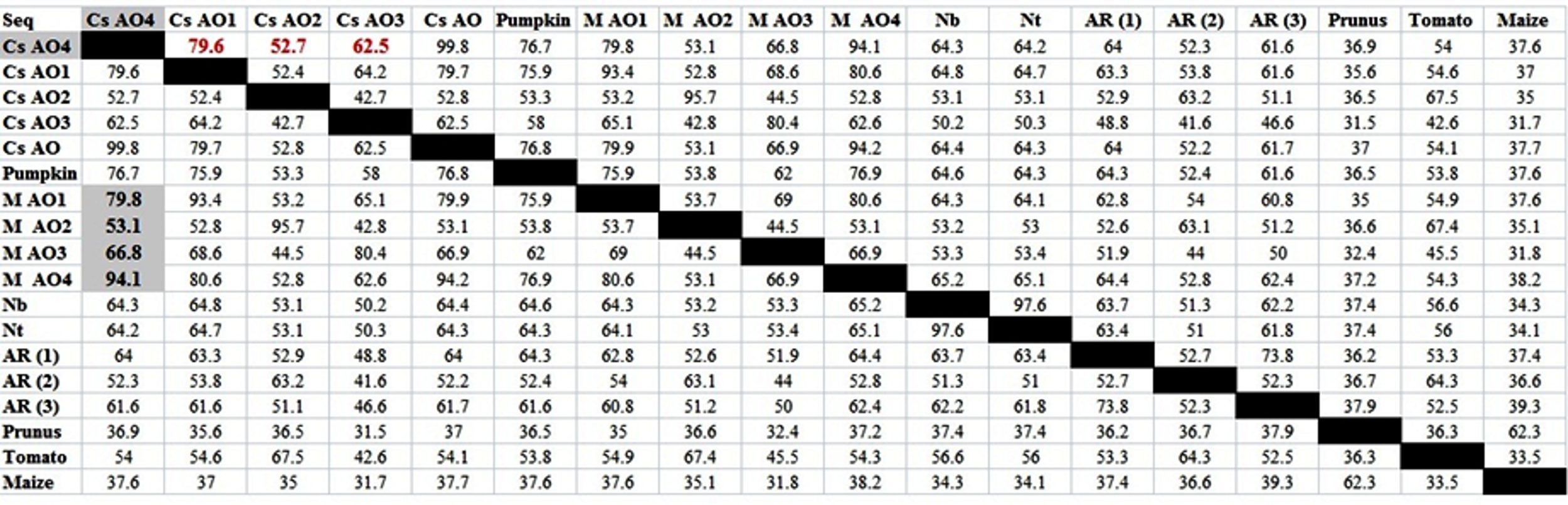

Supplement: S2 Fig — (A) Phylogenetic analysis of cucumber AO4 with other AO sequences taken from different plants by Neighbour-joining method in MEGA 6.0. Plants and their accession numbers used in study were mentioned in the phylogenetic tree. Bootstrap values were indicated at branches and evolutionary distance was shown below the figure; (B) Percent identity matrix of nucleotide sequences of AO from different plants. Sequence identity of CsAO4 with other cucumber homolog’s (CsAO1, CsAO2, and CsAO3) was shown in red letters; and with melon homolog’s (M AO1, M AO2, M AO3, and M AO4) was shown in grey shaded bold letters. Percent identity matrix was 1calculated using Bioedit software (version 7.1.3.0). CsAO1 (Cucumber AO1:XM_004144064), CsAO2 (Cucumber AO2:XM_004173026), CsAO3 (Cucumber AO3:XM_004144107), Cucumber AO (J04494), Pumpkin (D55677), M AO1 (Melon AO1:AF233593), M AO2 (Melon AO2:XM_008442414), M AO3 (Melon AO3:Y10226), M AO4 (Melon AO4:AF233594), Nb (N. benthamiana:HG938363), Nt (N. tabaccum:D43624), Arabidopsis [AR(1): AT5G21105, AR(2):AT4G39830, AR(3): AT5G21100], Prunus (KC152937), Tomato (AY971876), and Maize (EU973211). (TIF) [file pone.0163320.s002.tif]

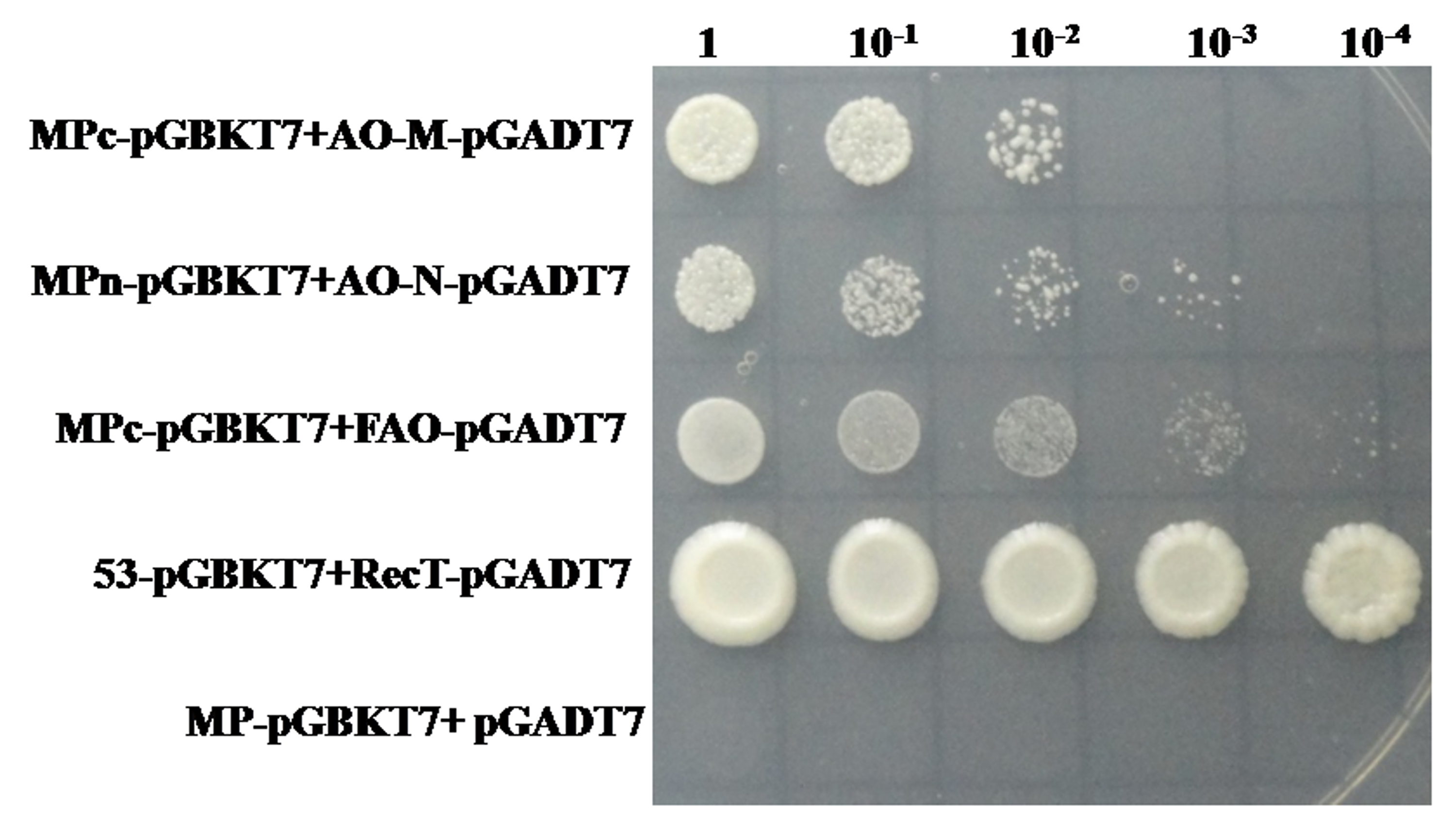

Supplement: S3 Fig — The figure showing strength of interaction of full length AO and its domains with MP domains as indicated by growth on selection plate along with positive and negative controls. (TIF) [file pone.0163320.s003.tif]

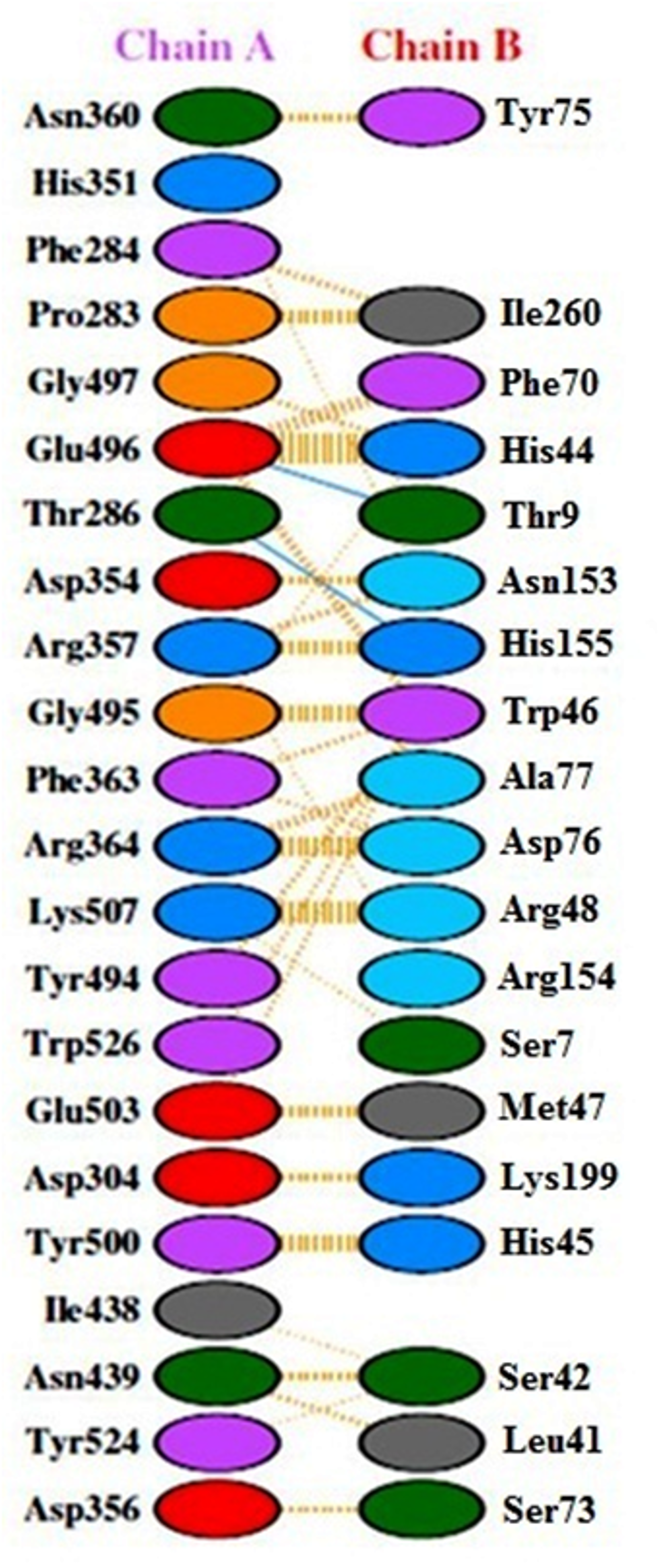

Supplement: S4 Fig — Based on the results of LigPlot+ interaction plot was generated showing hydrogen and hydrophobic interactions between residues. Chain A and Chain B denotes CsAO4 and CMV MP respectively. (TIF) [file pone.0163320.s004.tif]

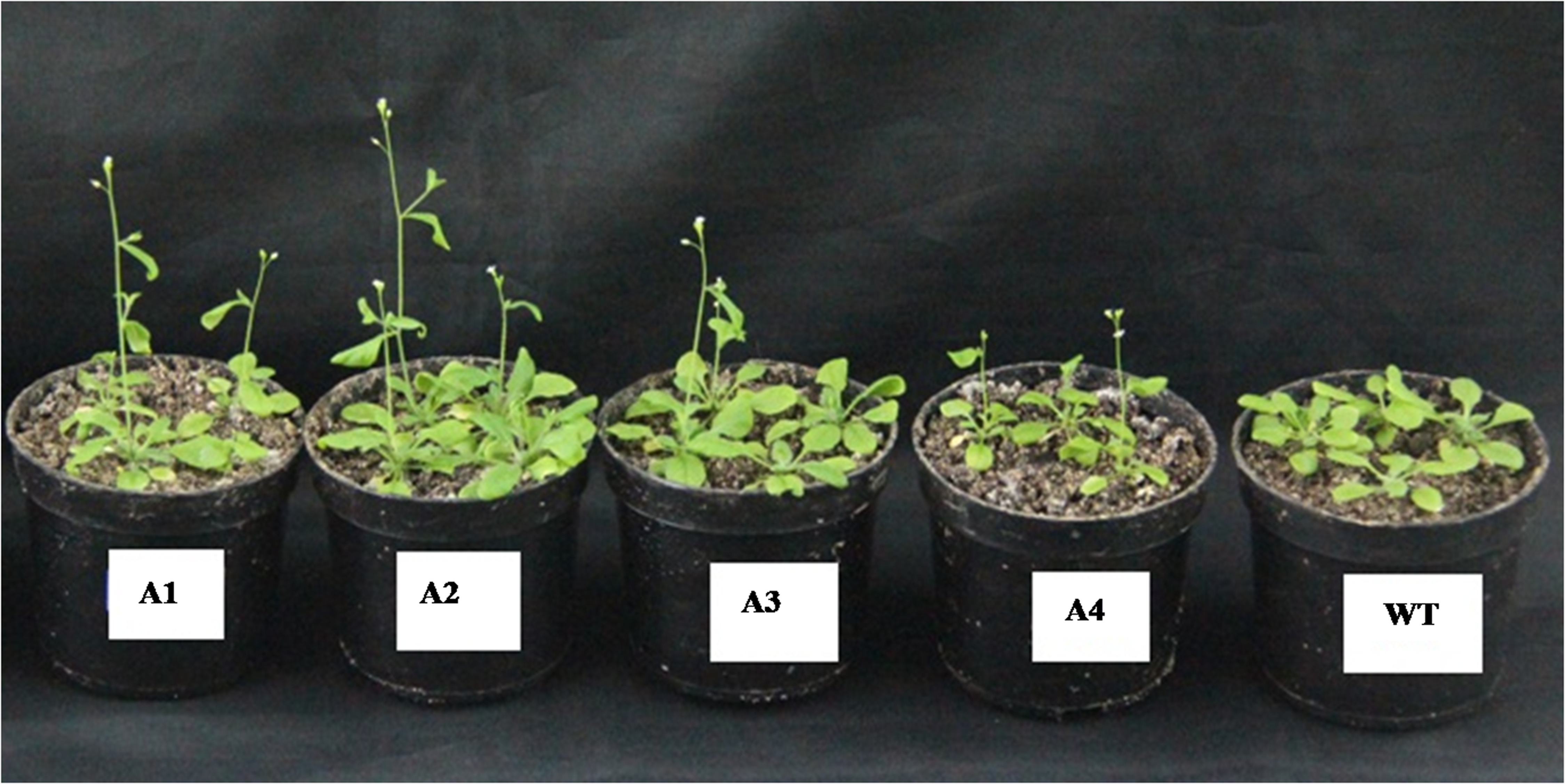

Supplement: S5 Fig — Early flowering observed in AO-overexpressing transgenic lines in comparison to wild type plants. Representative pictures of T2 plants (Four lines: A1, A2, A3, A4) and WT were shown. (TIF) [file pone.0163320.s005.tif]

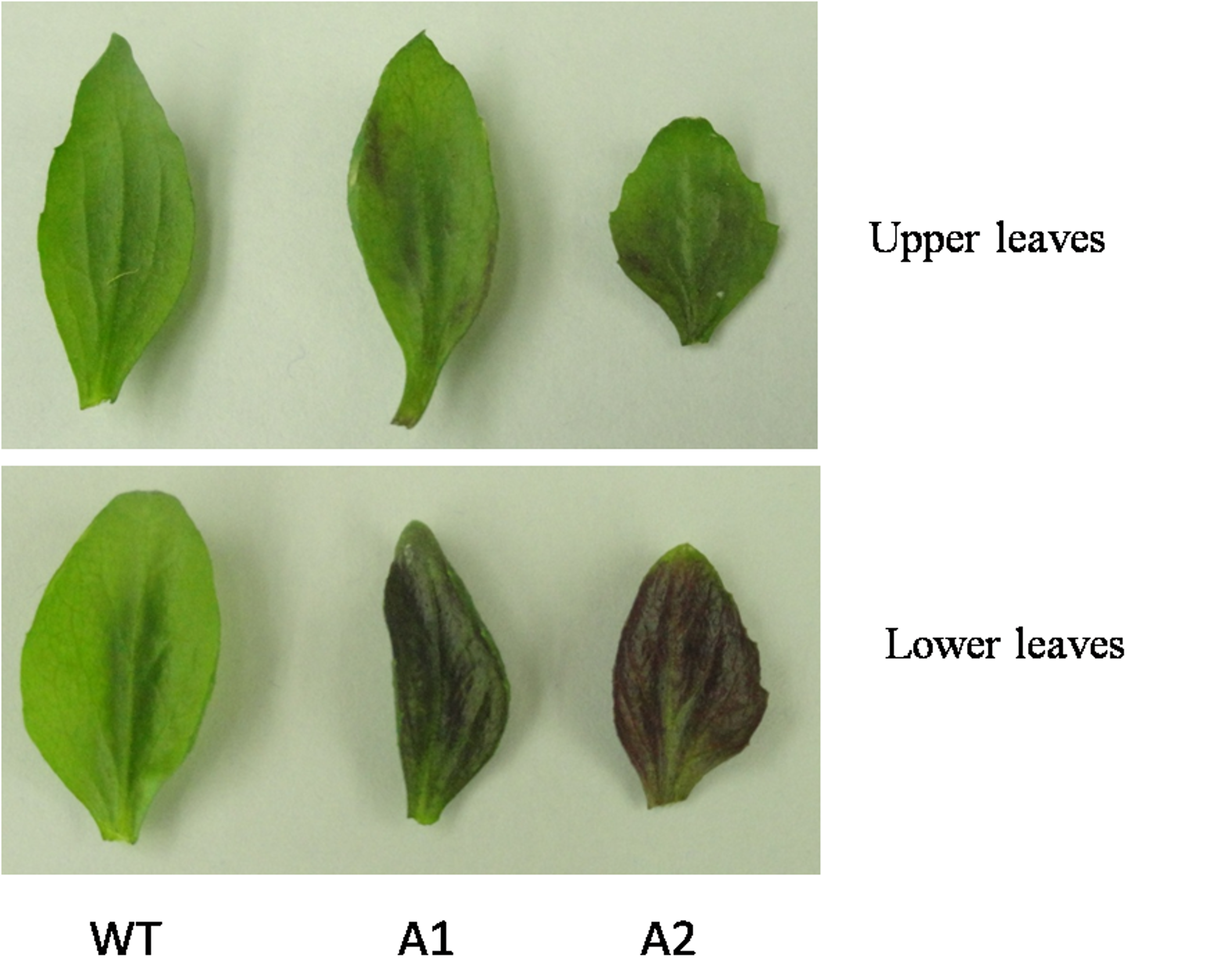

Supplement: S6 Fig — Anthocyanin pigmentation was observed in AO-overexpressing transgenic lines in comparison to wild type plants. Representative pictures of upper and lower leaves of CMV infected transgenic (two lines: A1, A2) and WT plants after two weeks were shown in the figure. (TIF) [file pone.0163320.s006.tif]

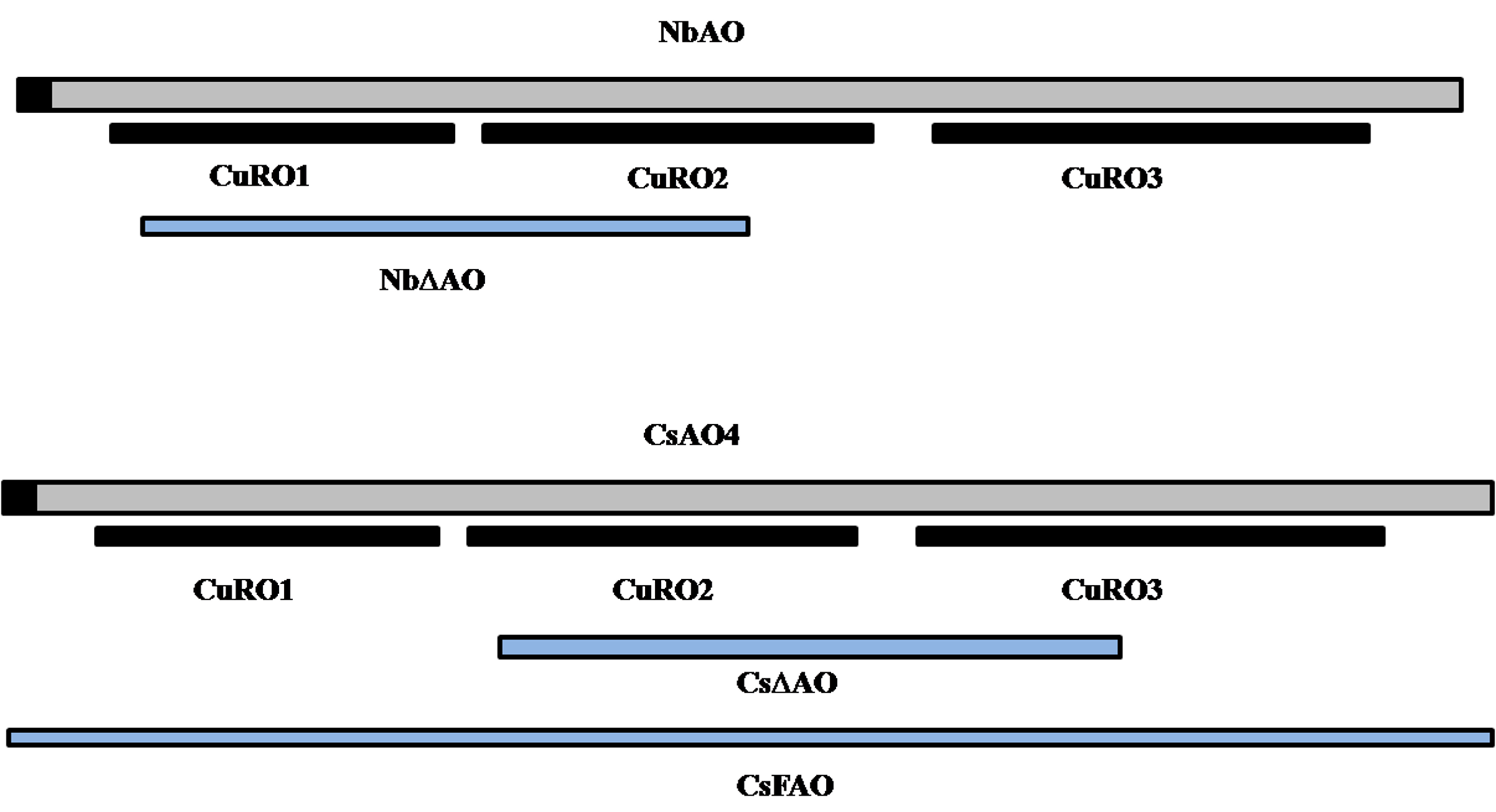

Supplement: S7 Fig — The figure showed regions of NbAO (NbΔAO) and CsAO4 (CsFAO and CsΔAO) indicated by blue bars and cupredoxin domain 1 (CuRO1), domain 2 (CuRO2) and domain 3 (CuRO3) indicated by solid black bars. (TIF) [file pone.0163320.s007.tif]

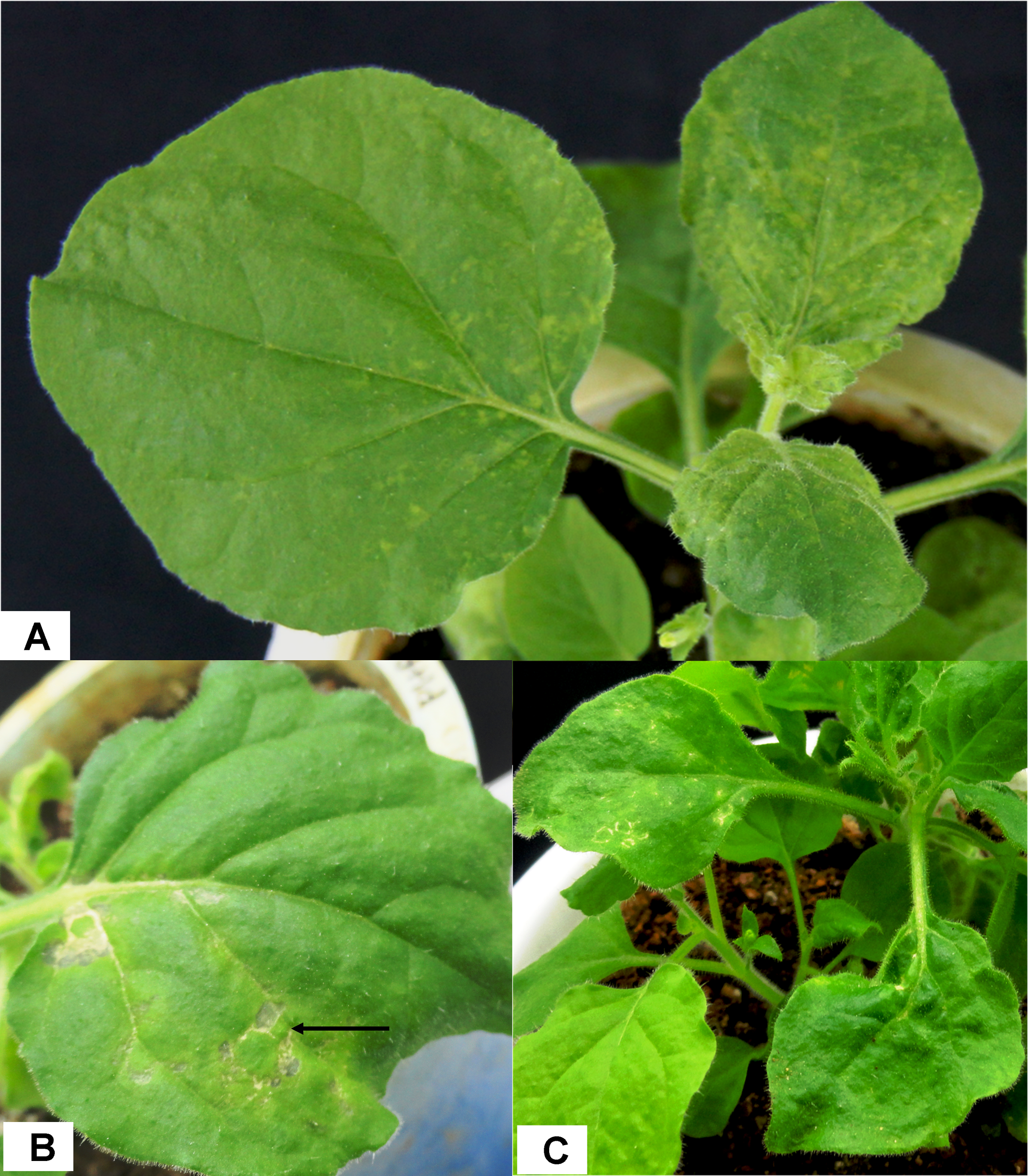

Supplement: S8 Fig — Phenotypic effect of NbAO knockdown was observed on plants. (A-B) Full length CsAO4-pTRV2 (CsFAO-pTRV2): developed chlorotic patches along with necrotic areas above infiltrated leaves; (C) Partial CsAO4-pTRV2 (CsΔAO-pTRV2): caused symptoms like leaf deformation, necrotic areas, and size reduction in new emerging leaves. (TIF) [file pone.0163320.s008.tif]

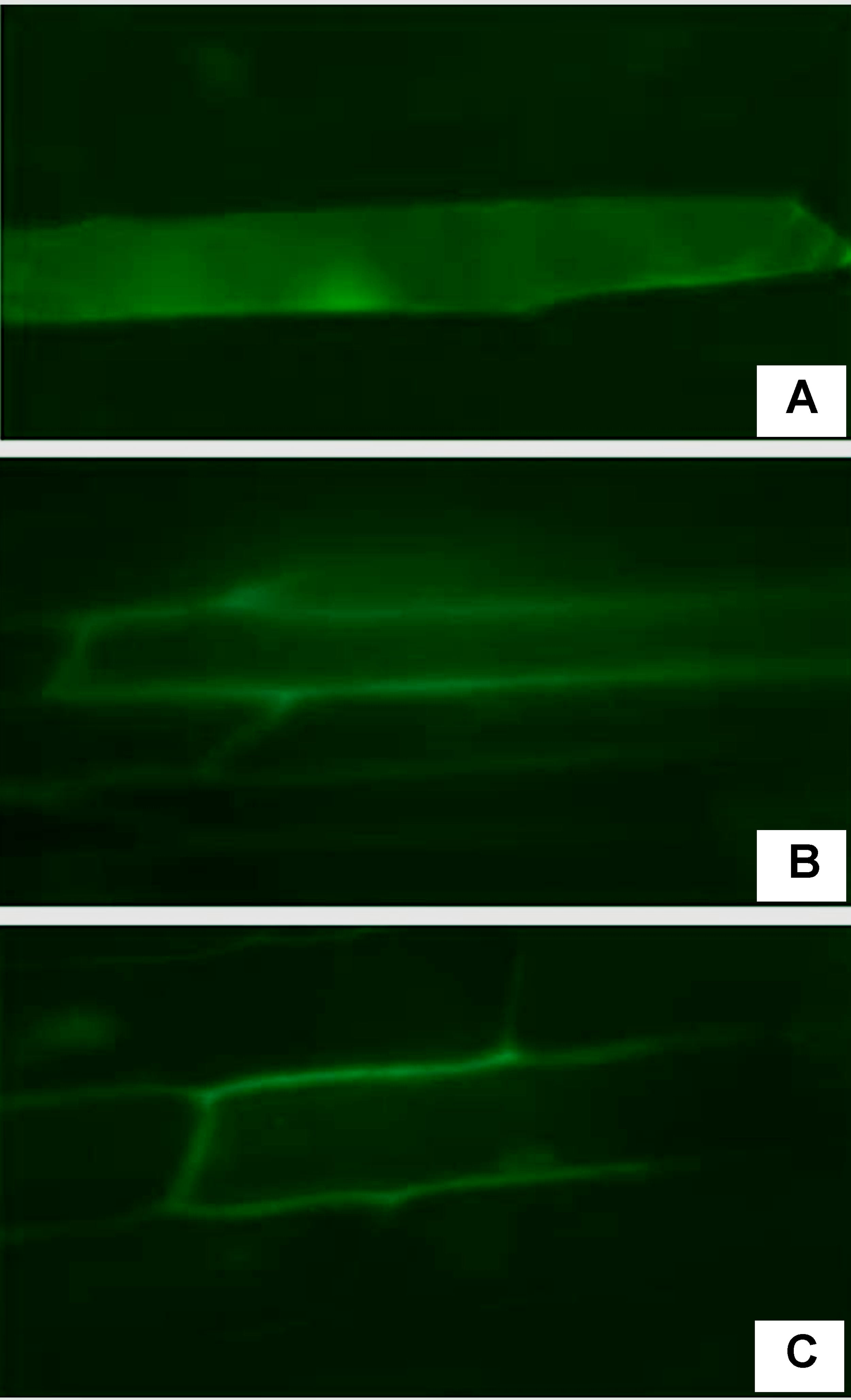

Supplement: S9 Fig — pCAMBIA1302 constructs were bombarded on onion epidermal peels by biolisitc method and GFP fluorescence was observed under fluorescent microscope. (A) Empty pCAMBIA1302 vector showing free GFP localization in cytoplasm and nucleus. (B) CMV MP-pCAMBIA1302 and (C) CsAO4-pCAMBIA1302 showing localization around cell wall region. (TIF) [file pone.0163320.s009.tif]
