# Supplementary material for: Movement Protein of Cucumber Mosaic Virus Associates with Apoplastic Ascorbate Oxidase
Source: PLoS One. 2016 Sep 26;11(9):e0163320. doi: 10.1371/journal.pone.0163320 (PMC5036820; doi:10.1371/journal.pone.0163320)
Supplement: S1 Table — (DOCX) [file pone.0163320.s010.docx]

**S1 Table.** Primers used during the study

|  | **Construct** | **Primers (5’→3’)** |
| --- | --- | --- |
| **For Yeast Two hybrid assay** | | |
|  | MP | F- CATATGGCTTTTCAAGGT |
|  |  | R- CCCGGGCTAAAGACCGTTAA |
|  | MPn | F- CATATGGCTTTCCAAGGTACCAGT |
|  |  | R- GAATTCGTTACTACACACGCTAGCAG |
|  | MPc | F- CATATGACGGATGCTGAGGGTTCTTT |
|  |  | R- GGATCCCTAAAGACCGTTAACCACCT |
|  | CsAO4 | F- TCTCCATGGCAAAAGTTGCAGATAAGCC |
|  |  | R- GACTAGTGGGTAATCGGGGATAGTTCT |
|  | AO-N | F- ATCCATATGGATGTTGAGTACATGTTTTGG |
|  |  | R- TTGAATTCTCCTTCTGGTGGATCCACTATC |
|  | AO-M | F- ATTCATATGGAAGAGATCAACTTATTGCTT |
|  |  | R- TTGGATCCGTTGGGGAGGTAATTGAGGA |
|  | AO-C | F- TCGAATTCGTTTACAAGTTCAATATGGGGG |
|  |  | R- TTGGATCCAACTCCTTCGGCAAACACAACT |
| **For VIGS assay** | | |
|  | CsFAO | F- TTGAATTCATGGCAAAAGTTGCAGATAAGC |
|  |  | R- TTGAGCTCGGGTAATCGGGGATAGTTCTT |
|  | Cs∆AO | F- TATGAATTCAGAAGTTGGTCTCAGCTCCAAAC |
|  |  | R- TCTGGATCCTCGGCGGTGGGTTGTTGATGTC |
|  | Nb∆AO | F- GGATCCGACGTGGAATATATTCATTGGTC |
|  |  | R- GAGCTCTTGTACTGAAAATGGTTGAACATA |
|  | NbAO | F- ATGGCTTCCTTAGGCTTCTTGTTC |
|  |  | R- TTAATTATGCTTGTTACTCATCAAC |
|  | TRV1 | F- CTTGAAGAAGAAGACTTTCGAAGT |
|  |  | R- GATAACAACACAGACAAACCATCCAC |
|  | NbActin | F- CATACAAGCCAACCACGGCCTCC |
|  |  | R- GCGTGGATATGTCCTGCGGGTA |
|  | NbPDS | F- AGGAATTCCATAAACCCTGACGAGCTTTCG |
|  |  | R- TTGAGCTCGTGTACAACGCTAATTCAGCG |
| **For Overexpression in pCAMBIA1302** | | |
|  | CsAO4-pCAMBIA1302 | F- TCTCCATGGCAAAAGTTGCAGATAAGCC |
|  |  | R- GACTAGTGGGTAATCGGGGATAGTTCT |
|  | Hygromycin | F- CATACAAGCCAACCACGGCCTCC |
|  |  | R- GCGTGGATATGTCCTGCGGGTA |
| **For Real time PCR** | | |
|  | CsAO | F- GCCATTTAGGGATGCAAAGA |
|  |  | R- GGTTTGGAGCTGAGACCAAC |
|  | CMV CP | F- ACCCTGAAACCGCCTGAAAT |
|  |  | R- TCCGAACTGTAACCCACACG |
|  | 18s RNA | F- TCTGCCCGTTGCTCTGATG |
|  |  | R- TCACCCGTCACCACCATAG |
|  | Cucumber Actin | F- GGCAGTGGTGGTGAACA |
|  |  | R- CTGGTATCGTGCTGGATT |

Underlined regions indicate Restriction sites
